# The default methods in the 2019 Refinement drastically reduce estimates of global carbon sinks of harvested wood products

**DOI:** 10.1186/s13021-021-00200-8

**Published:** 2021-12-11

**Authors:** Chihiro Kayo, Gerald Kalt, Yuko Tsunetsugu, Seiji Hashimoto, Hirotaka Komata, Ryu Noda, Hiroyasu Oka

**Affiliations:** 1 Graduate School of Agriculture, Tokyo University of Agriculture and Technology, 3-5-8 Saiwai-cho, Fuchu, Tokyo 183-8509 Japan; 2 Institute of Social Ecology, University of Natural Resources and Life Sciences, Schottenfeldgasse 29, 1070 Vienna, Austria; 3 Graduate School of Agricultural and Life Sciences, The University of Tokyo, 1-1-1 Yayoi, Bunkyo-ku, Tokyo, 113-8657 Japan; 4 College of Science and Engineering, Ritsumeikan University, 1-1-1 Noji-higashi, Kusatsu, Shiga 525-8577 Japan; 5 Forest Research Department, Hokkaido Research Organization, 1-10 Nishikagura, Asahikawa, Hokkaido 071-0198 Japan; 6 Institute of Wood Technology, Akita Prefectural University, 11-1 Kaieizaka, Noshiro, Akita 016-0876 Japan; 7 Forestry Division, Japan International Research Center for Agricultural Sciences, 1-1 Ohwashi, Tsukuba, Ibaraki 305-8686 Japan

**Keywords:** Harvested wood products, Carbon stocks, Carbon removals, Stock-change approach, Atmospheric flow approach, Production approach, Guidelines for National Greenhouse Gas Inventories, Kyoto Protocol, Paris Agreement, United Nations Framework Convention on Climate Change

## Abstract

**Background:**

The stock dynamics of harvested wood products (HWPs) are a relevant component of anthropogenic carbon cycles. Generally, HWP stock increases are treated as carbon removals from the atmosphere, while stock decreases are considered emissions. Among the different approaches suggested by the Intergovernmental Panel on Climate Change (IPCC) for accounting HWPs in national greenhouse gas inventories, the production approach has been established as the common approach under the Kyoto Protocol and Paris Agreement. However, the 24th session of the Conference of the Parties to the United Nations Framework Convention on Climate Change decided that alternative approaches can also be used. The IPCC has published guidelines for estimating HWP carbon stocks and default parameters for the various approaches in the 2006 Guidelines, 2013 Guidance, and 2019 Refinement. Although there are significant differences among the default methods in the three IPCC guidelines, no studies have systematically quantified or compared the results from the different guidelines on a global scale. This study quantifies the HWP stock dynamics and corresponding carbon removals/emissions under each approach based on the default methods presented in each guideline for 235 individual countries/regions.

**Results:**

We identified relatively good consistency in carbon stocks/removals between the stock-change and the atmospheric flow approaches at a global level. Under both approaches, the methodological and parameter updates in the 2019 Refinement (e.g., considered HWPs, starting year for carbon stocks, and conversion factors) resulted in one-third reduction in carbon removals compared to the 2006 Guidelines. The production approach leads to a systematic underestimation of global carbon stocks and removals because it confines accounting to products derived from domestic harvests and uses the share of domestic feedstock for accounting. The 2013 Guidance and the 2019 Refinement reduce the estimated global carbon removals under the production approach by 15% and 45% (2018), respectively, compared to the 2006 Guidelines.

**Conclusions:**

Gradual refinements in the IPCC default methods have a considerably higher impact on global estimates of HWP carbon stocks and removals than the differences in accounting approaches. The methodological improvements in the 2019 Refinement halve the global HWP carbon removals estimated in the former version, the 2006 Guidelines.

**Supplementary Information:**

The online version contains supplementary material available at 10.1186/s13021-021-00200-8.

## Background

Combating the climate change caused by increasing concentrations of greenhouse gases (GHG) in the atmosphere is among humanity’s biggest challenges. At the 21st session of the Conference of the Parties (COP21) to the United Nations Framework Convention on Climate Change (UNFCCC), held in Paris in 2015, participant countries adopted the Paris Agreement, which set global reduction targets for 2020 onward. The participants agreed to undertake ambitious efforts to maintain the global temperature increase below 2 °C during this century [1]. Monitoring these efforts requires the development of standardized accounting frameworks to quantify the human impact on the global carbon cycle.

Forests play a significant role in the global carbon cycle. According to the Global Carbon Project [2], the global land carbon sink, including forest biomass, accounted for the removal of 3.2 ± 0.6 gigatons of atmospheric carbon per year (GtC year^−1^) during 2009–2018, equivalent to 34% of the annual carbon emissions from fossil fuel combustion and industrial processes. The widespread use of wood products in society and their discardment after their use periods are also a human-induced change to the global carbon cycle. With forests and stocks of harvested wood products (HWPs) considered as separate carbon pools, carbon stock increases in HWPs correspond to carbon removals from the atmosphere whereas stock decreases correspond to emissions in the HWP pool. While carbon is actually just transferred from the forest to the HWP pool, this transfer is modeled as a decrease in forest carbon stocks and an increase in the carbon stock of HWPs under the GHG accounting framework of the UNFCCC; forests and HWP pools are thus communicating vessels that are regionally dispersed due to international trade with HWPs. Although what is relevant in terms of climate change is the global net carbon balance of wood (determined by the sum of carbon stock changes in forest and HWP pools), a separate accounting of forests and HWP pools is necessary and indispensable for understanding global carbon dynamics. Historically, global carbon stocks in HWPs have grown considerably, corresponding to annual carbon removals in the range of 0.03 to 0.3 GtC year^−1^ [3–7] (net transfers from forests to HWP pools).

An intricate issue in the context of HWP accounting is international trade. International supply chains are highly complex and, depending on the chosen system boundaries, the estimated results of accounting frameworks may differ considerably, particularly at the national level. Over the past two decades, several approaches for accounting HWPs using different system boundaries have been proposed, and their implications for climate change policy discussed. Specifically, Brown et al. [8] and Lim et al. [9] proposed the stock-change approach (SCA), atmospheric flow approach (AFA), and production approach (PA), while Skog et al. [10] and Pingoud and Wagner [11] improved the SCA. These studies contributed to the 2006 Intergovernmental Panel on Climate Change (IPCC) Guidelines for National Greenhouse Gas Inventories (hereafter, the 2006 Guidelines) [12].

Previous studies estimated HWP carbon stocks and their changes using the SCA for specific countries (e.g., [10, 11, 13, 14]) or at the global level [5, 6], while the PA has so far been applied only at the national level (e.g., [15, 16]).

Since the SCA, AFA, and PA differ with respect to system boundaries, particularly in the accounting of traded wood products, the results of the different approaches can vary considerably for individual countries, especially those with high trade flows. Consequently, several previous studies have compared the SCA, AFA, and/or PA for specific countries [6, 17–25] or at the global level [7], revealing that different approaches have markedly different implications for the countries that import HWPs compared to those that export them.

During the 7th session of the Conference of the Parties serving as the meeting of the Parties to the Kyoto Protocol (CMP7), held in Durban in 2011 [26], the parties agreed to use the PA, which only includes the HWPs produced domestically from a country’s forests in the national GHG inventory but disregards where the HWPs are actually used, as the common accounting approach for Annex I Parties for the second commitment period of the Kyoto Protocol from 2013 to 2020. Accordingly, a modified version of the PA as per the Kyoto Protocol (PA13) was included in the 2013 Revised Supplementary Methods and Good Practice Guidance Arising from the Kyoto Protocol (hereafter, the 2013 Guidance) [27]. The 2013 Guidance was provided to meet the rules for the treatment of Land Use, Land-Use Change and Forestry (LULUCF) in the second commitment period agreed by CMP7 after negotiations between nations and, thus, the PA13 was not a purely scientific improvement compared to the PA in the 2006 Guidelines. Recent studies have used the PA13 to estimate HWP carbon stocks and their changes both for individual countries [25, 28–34] as well as globally [4].

Determining the most appropriate approach has thus become a crucial issue with respect to the Paris Agreement as well. According to the decision adopted at the COP24 held in Katowice in 2018, countries can choose any approach when accounting for removals/emissions for HWPs for their nationally determined contributions (NDCs) [35, 36]. Meanwhile, although countries are also free to choose any approach in their national GHG inventory reporting under the Paris Agreement, a country not using the PA is requested to provide results of removals/emissions for HWPs using the PA as supplementary information [36, 37]. For national GHG inventories, the 2006 Guidelines [12] for the SCA, AFA, and PA were improved under the 2019 Refinement to the 2006 IPCC Guidelines for National Greenhouse Gas Inventories (hereafter, the 2019 Refinement) [38] for the SCA19, AFA19, and PA19. A recent study included the 2019 Refinement to estimate the HWP carbon stocks and their changes in New Zealand [39].

In sum, carbon accounting approaches are described in the three IPCC guidelines [12, 27, 38]. While their basic principles remain the same, the specific implementation and default methods and parameters (e.g., assumed product lifetimes, carbon conversion factors) in the 2006 Guidelines have been revised in the 2013 Guidance and the 2019 Refinement, leading to different results for the estimated carbon stocks, both nationally and globally. These differences may hinder a proper understanding of HWP contributions to the global carbon cycle. Although the basic approaches have been discussed and compared thoroughly in the literature, no study has thus far systematically quantified and compared the implications of the different IPCC guidelines at the national and global levels.

Aiming to fill this research gap, this study quantifies the carbon stocks dynamics and corresponding carbon removals/emissions of HWPs by deriving global estimates, using each default method under each accounting approach described in the three IPCC guidelines for individual countries. Further, it discusses the implications of the different IPCC guidelines.

## Results

### Global carbon stocks and removals of HWPs

**Fig. 1:**
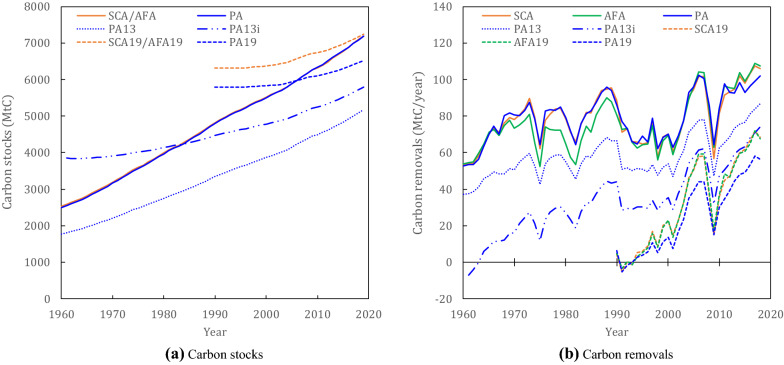
Global carbon stocks and carbon removals for harvested wood products (HWPs). “SCA,” “AFA,” and “PA” denote the default (Tier 1) method for the stock-change approach, atmospheric flow approach, and production approach, respectively, according to the 2006 Guidelines [12]. “PA13” and “PA13i” denote the default (Tier 2) method for the production approach assuming that the initial years in carbon stocks are 1900 and 1961, respectively, according to the 2013 Guidance [27]. “SCA19,” “AFA19,” and “PA19” denote the default (Tier 1) method for the stock-change approach, atmospheric flow approach, and production approach, respectively, according to the 2019 Refinement [38]

The global total of carbon stocks and carbon removals of HWPs, determined using the default methods under different accounting approaches according to IPCC guidelines, are shown in Fig. 1. Following the various IPCC guidelines results in different amounts of carbon stocks. The global carbon stocks based on the SCA and AFA are identical, as these approaches only differ in how they derive carbon removals from stock changes. The PA also results in similar global stocks when applying the method described in the 2006 Guidelines. By contrast, the global carbon stocks resulting from the PA13 according to the 2013 Guidance, are only around 72% of those when applying the PA. The SCA19, AFA19, and PA19, according to the 2019 Refinement, show developments in carbon stocks that differ considerably from those calculated using the previous guidelines. The values of carbon stocks under the PA13i are between those for the PA13 and PA19. In relation to the carbon stocks in living biomass in global planted forests in 2019 [40, 41], the HWP carbon stocks fluctuated between a minimum of 53% for the PA13 and a maximum of 75% for the SCA19/AFA19. These differences in carbon stock dynamics translate into the following ranges for global carbon removals of HWPs: the global carbon removals in 2018 were 106 megatons of carbon per year (MtC year^−1^) under the SCA and 107 MtC year^−1^ under the AFA. The PA results deviated from the SCA and AFA, particularly in the 2010s, amounting to 102 MtC year^−1^ in 2018. The carbon removals under the SCA19 and AFA19 were significantly smaller, at 68 MtC year^−1^ in 2018. The results for the PA13, PA13i, and PA19 were 87 MtC year^−1^, 74 MtC year^−1^, and 57 MtC year^−1^, respectively, in 2018. These results correspond to 85%, 73%, and 55%, respectively, of the PA. In relation to the global carbon emissions from fossil fuel combustion and industrial processes [2], the HWP removals in 2018 fluctuated between a minimum of 0.6% under the PA19 and a maximum of 1.1% under the AFA, while these results are equivalent to 3.1–6.0% of the carbon emissions from global fires (on average during 2000–2019) [42]. These values further correspond to 1.6–3.1% of terrestrial carbon sinks [2] and 47.9–90.9% of carbon removals of global planted forests [40, 41].

### Examples of national carbon stocks and removals of HWPs

**Fig. 2:**
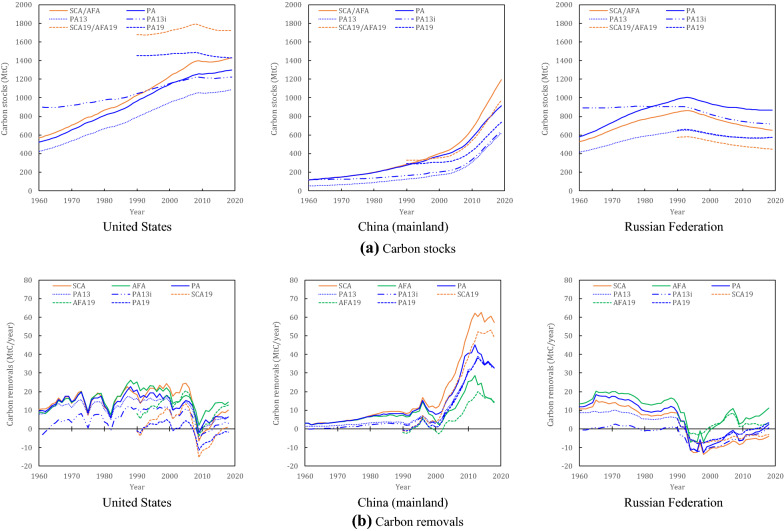
National carbon stocks and carbon removals for harvested wood products (HWPs). “SCA,” “AFA,” and “PA” denote the default (Tier 1) method for the stock-change approach, atmospheric flow approach, and production approach, respectively, according to the 2006 Guidelines [12]. “PA13” and “PA13i” denote the default (Tier 2) method for the production approach assuming that the initial years in carbon stocks are 1900 and 1961, respectively, according to the 2013 Guidance [27]. “SCA19,” “AFA19,” and “PA19” denote the default (Tier 1) method for the stock-change approach, atmospheric flow approach, and production approach, respectively, according to the 2019 Refinement [38]

Figure 2 shows the carbon stocks and carbon removals of HWPs in China (excluding Hong Kong, Macao, and Taiwan), the United States, and the Russian Federation, which are the world’s top three holders of HWP carbon stocks. For all default methods, the United States contributes the most to global HWP carbon stocks. The US share of global HWP stocks ranged from 18% under the PA to 24% under the SCA19/AFA19 in 2019. Meanwhile, for the carbon removals, due to a steeper increase in its HWP stocks, China contributed the most to global HWP carbon removals, with the values ranging from 13% under the AFA to 72% under the SCA19 in 2018. The SCA and SCA19 are the most advantageous for China because they estimate the actual HWP consumption, whereas the PA and AFA attribute carbon removals to producing and exporting countries. By contrast, the United States and the Russian Federation—both large HWP exporters—have the highest carbon removals based on the AFA and AFA19. The national carbon stocks and removals of HWPs for the various countries are provided in Additional files 1, 2, 3, 4, 5, 6, 7, 8.

## Discussion

### Global perspective

Regarding the accounting approaches in the 2006 Guidelines, the results of the SCA and AFA show identical global carbon stocks (Fig. 1a). If all activity data at the global level are consistent, identical results would also be achieved for global carbon removals. In reality, however, we find differences, particularly before the 1990s (Fig. 1b), which can be attributed to inconsistencies between the import and export volumes at the global level [43]. Since the AFA uses trade data for all wood-based materials (see Eq. (2)), there is a small difference between the SCA and AFA results. Activity data for newly independent countries after 1961 also lead to trade inconsistencies, because the default method suggested in the 2006 Guidelines, which uses the rate of annual change in each dataset for the old country that a new country is part of, ignores the balance between import and export volumes for this period (see section "Implementation of the IPCC guidelines"). The carbon removals under the PA differ slightly from those under the two aforementioned approaches, particularly in the 2010s, owing to the fact that the former confines HWP accounting to products originating from domestic forests. Specifically, to estimate the inflow to carbon stocks in HWPs derived from domestic harvest (see Eq. (9)), the proportional share of the domestic feedstock is used (see Eq. (10)). We surmise this difference is because the share of the domestic feedstock is assumed to be 0 (see section "Implementation of the IPCC guidelines") in several countries that actually have negative values for the share, due to an error in the trade database [43].

The results for the SCA19 and AFA19 in the 2019 Refinement are similar to those of the SCA and AFA in the 2006 Guidelines because the inconsistencies between globally aggregated import and export volumes are relatively small after 1990. However, the carbon stocks and removals in the PA19 are significantly lower than those in the SCA19 and AFA19. In addition to the aforementioned arguments for the PA, the differences are also derived from handling exported feedstock originating from domestic forests. Unlike the 2006 Guidelines (see Eq. (10)), the 2019 Refinement (and the 2013 Guidance) suggested subtracting feedstock exports from feedstock production in the numerator for the share of domestic feedstock (i.e., industrial roundwood, wood pulp, and recovered paper) (see Eqs. (11)–(13) and Table 1). This exclusion of exported feedstock from the share of domestic feedstock means that the PA19 further reduces the calculated carbon stocks and removals compared to the SCA19 and AFA19. Hence, the exported feedstock is neither accounted for in the carbon stocks of the exporter country nor in those of the importer country, resulting in omissions of the corresponding stocks.

**Table 1:** Overview of the accounting approaches and the IPCC guidelines for harvested wood products (HWPs)

Accounting approach		SCA	AFA	PA	PA13 (PA13i)	SCA19	AFA19	PA19
IPCC guidelines		2006 Guidelines (Tier 1)	2006 Guidelines (Tier 1)	2006 Guidelines (Tier 1)	2013 Guidance (Tier 2)	2019 Refinement (Tier 1)	2019 Refinement (Tier 1)	2019 Refinement (Tier 1)
Considered HWPs		Sawnwood, wood-based panels, paper and paperboard, other industrial roundwood	Sawnwood, wood-based panels, paper and paperboard, other industrial roundwood	Sawnwood, wood-based panels, paper and paperboard, other industrial roundwood	Sawnwood, wood-based panels, paper and paperboard	Sawnwood, wood-based panels, paper and paperboard	Sawnwood, wood-based panels, paper and paperboard	Sawnwood, wood-based panels, paper and paperboard
HWP activity data		FAOSTAT						
Decay function		First-order decay function						
Half-life (years)	Sawnwood	30	30	30	35	35	35	35
	Wood-based panels	30	30	30	25	25	25	25
	Paper and paperboard	2	2	2	2	2	2	2
	Other industrial roundwood	30	30	30				
Inflow to carbon stocks		Consumed wood	Consumed wood	Harvested wood	Harvested wood	Consumed wood	Consumed wood	Harvested wood
Initial year for carbon stocks		1900	1900	1900	1900 (1961)	1990	1990	1990
Exports and imports for the AFA and AFA19			All wood-based materials				Feedstock for HWPs	
Share of domestic feedstock for the PA, PA13, and PA19	Sawnwood, wood-based panels, and other industrial roundwood			Production (using IRW, WCP, and WR)	Production minus exports (using IRW)			Production minus exports (using IRW)
	Paper and paperboard				Production minus exports (using IRW and WP)			Production minus exports (using IRW, WP, and RP)
Carbon conversion factors	Industrial roundwood, sawnwood, wood chips, wood residues, etc. (tC/m^3^)	0.225 (temperate) 0.295 (tropical)	0.225 (temperate) 0.295 (tropical)	0.225 (temperate) 0.295 (tropical)	0.229 (aggregate)	0.229 (aggregate)	0.229 (aggregate)	0.229 (aggregate)
	Wood-based panels (tC/m^3^)	0.294	0.294	0.294	0.269	0.269	0.269	0.269
	Paper and paperboard (tC/t)	0.450	0.450	0.450	0.386	0.386	0.386	0.386
	Wood pulp (tC/t)		0.450		0.386		0.417	0.417
	Wood charcoal (tC/t)		0.765				0.765	

“SCA,” “AFA,” and “PA” denote the stock-change approach, atmospheric flow approach, and production approach, respectively. “IRW,” “WCP,” “WR,” “WP,” and “RP” denote industrial roundwood, wood chips and particles, wood residues, wood pulp, and recovered paper, respectively

The main reason for these considerable differences between the SCA and AFA and their 2019 counterparts (i.e., the SCA19 and AFA19) is a change in the starting year for carbon stock calculations. According to the default (Tier 1) method in the 2006 Guidelines, the starting year is 1900, whereas it is 1990 according to the 2019 Refinement, and the method for estimating the 1990 stocks according to the 2019 Refinement (see Eq. (8)) results in higher global stocks than the backcasting method suggested in the 2006 Guidelines (see Eq. (7)). Furthermore, the 2019 Refinement proposed different conversion factors for the density and carbon fraction of each HWP, which are relatively lower than those in the 2006 Guidelines (see Table 1). Additionally, the default (Tier 1) method in the 2019 Refinement does not consider “other industrial roundwood” (see the categories in the FAOSTAT or the Food and Agriculture Organization of the United Nations’ database) [43] in the stock calculation because the wood products in this category (e.g., transmission poles and roundwood used directly in the construction of buildings) have less uniform characteristics than the considered wood products (i.e., sawnwood, wood-based panels, and paper and paperboard) [38], whereas the default method in the 2006 Guidelines includes this category (Table 1). Note that the 2019 Refinement suggests using the Tier 3 method for calculating this category, and the method requires a more sophisticated representation of the category [38]. These revisions contribute to slower net stock accumulation since 1990. Consequently, the carbon removals according to the SCA19 and AFA19 are significantly lower than those according to the SCA and AFA.

The differences between the results obtained using the PA, PA13, and PA19 arise from the reduced default carbon conversion factors in the more recent guidelines, as well as methodological differences. Specifically, exported feedstock is excluded from the share of domestic feedstock in the PA13 and PA19 (see Eqs. (11)–(13) and Table 1). In addition, the more elaborate and complicated the equations for the share of domestic feedstock in the later guidelines (see Eqs. (10)–(13)), the more countries and years whose share of the domestic feedstock is assumed to be 0 because of a negative value for the share increase (see Additional files 4, 5, and 8), leading to decreased carbon removals. The PA19 further reduces carbon removals because there are differences in the initial year for carbon stocks (see Table 1). The PA13, PA13i, and PA19 use different initial years for carbon stocks, namely, 1900, 1961, and 1990, respectively. We find that the later the initial year is, the larger the initial carbon stocks are and, thus, the lower the resulting carbon removals (because higher stocks in the early years result in higher carbon outflows from the stocks in later years and, thus, lower net carbon stock increases; see Fig. 1). The 2019 Refinement suggests using later initial years (e.g., 1990) rather than earlier ones (e.g., 1961) to avoid the overestimation of carbon removals. This is because the coverage of statistical data on wood products has increased over time due to developing wood industries which exceed a certain threshold size to contribute statistics and, thus, the statistics have become more comprehensive. As a result, increasing trends in long time series data on wood products (e.g., since 1961) might partly reflect progressive improvements in the statistics rather than their actual changes. This implies that using earlier years (e.g., 1961) for initial carbon stocks might lead to biased estimates and result in the overestimation of subsequent carbon stock increases (i.e., carbon removals) in HWPs [38]. This suggestion agrees with our results of the PA13i and PA19. Consequently, the differences in the initial year of carbon stocks, as well as the method for estimating the carbon stocks in the initial year, result in significantly different carbon stocks and removals.

### National level perspective

At the national level, the different accounting approaches lead to highly different results among countries due to the widely varying trade balances. However, the three IPCC guidelines also lead to different results at the national level, even in the same accounting approach.

Among the three IPCC guidelines, the 2019 Refinement (SCA19, AFA19, and PA19) yields the highest carbon stocks in the United States and the lowest carbon stocks in the Russian Federation. For China, the influence of the methodological revisions in the 2019 Refinement on carbon removals is rather small in recent years. These differences between countries are due to the magnitudes of carbon inflows between 1990 and 1994, which determine the carbon stocks in the initial year according to the 2019 Refinement (see Eq. (8)). From 1990 to 1994, the largest carbon inflow in the world was recorded by the United States, while the Russian Federation’s inflow decreased rapidly after the dissolution of the Soviet Union in 1991 [43]. For China, the carbon inflow remained low, since drastic economic growth had yet to occur.

Regarding carbon removals, the United States obtained negative values (i.e., carbon emissions) after 2008 under the SCA19 and PA19, reflecting the impact of the global economic recession due to the 2007–2008 global financial crisis. The results for the Russian Federation show declining stocks (i.e., carbon emissions) for almost every year since the early 1990s and under all approaches, except for the AFA and the AFA19. For China, the carbon removals vary by a factor of more than 3, depending on whether the SCA/SCA19 or the AFA/AFA19 is applied. However, the differences between the different versions of each approach are relatively small.

For the PA, PA13, PA13i, and PA19, the carbon removals in China show slight differences and almost the same values in 2018. Meanwhile, the carbon removals for both the United States and the Russian Federation can be positive or negative over the past 10 to 20 years, depending on the applied approach. The PA19 proved disadvantageous for the United States in 2018, whereas it was advantageous for the Russian Federation. This opposite trend between the two countries results from the differences in the magnitudes of carbon stocks in the initial year and the subsequent carbon inflows of HWPs.

### Comparison with previous studies

We compare our results for global HWP carbon removals with the results of previous studies. Compared with the global removals reported by Lauk et al. [5] using the SCA between 1960 and 2008 and following the 2006 Guidelines, the two sets of results match over this period. However, Pingoud et al. [6] reported global carbon removals of 30–60 MtC year^−1^ between 1961 and 2000 using the SCA, which are lower than our results for the same approach and period, at 54–95 MtC year^−1^. We surmise that the differences are due to the variations in parameters for half-life (e.g., Pingoud et al. [6] assumed 1 year for paper and paperboard, while our study adopted the IPCC default values for half-lives, that is, 2 years for paper and paperboard; see Table 1), conversion factors (e.g., Pingoud et al. [6] applied densities for solid wood products lower than the IPCC default values; and the former values were the same as densities for temperate species in the 2006 Guidelines, while the latter values were considered for both temperate and tropical species), and the extrapolation of HWP data before 1961. Our results for global removals of 89 MtC year^−1^ for the SCA in 1990 are lower than those of 139 MtC year^−1^ reported by Winjum et al. [7]. Note that the SCA in Winjum et al.’s study [7] used methods and parameters different from the SCA in this study, which applied the 2006 Guidelines. The differences can be explained by the fact that Winjum et al. [7] did not consider the carbon emissions from the decay of HWPs stored prior to 1961 and applied half-life values that were longer than those used in this study. By comparison, under the PA13, our results for global removals during 1961–2015 (38–78 MtC year^−1^) are within the range reported by Johnston and Radeloff [4] (32–91 MtC year^−1^).

## Conclusions

We quantified carbon stocks and carbon removals for HWPs at the national and global levels under different accounting approaches and IPCC guidelines, and discussed the implications of using these different guidelines on carbon stocks and removals.

The default methods in the stock-change approach and the atmospheric flow approach result in highly consistent global carbon removal values, if calculated according to the same IPCC guidelines. Minor deviations are due to trade data inconsistencies. However, the comparison between the results according to the default method in the 2006 Guidelines (SCA and AFA) and those according to the 2019 Refinement (SCA19 and AFA19) revealed significant differences, with the carbon removals under the SCA19 and AFA19 being one-third lower than those under the SCA and AFA for 2018. This difference primarily originates from the different starting years for the carbon stock calculation and the methodological differences in the calculation of initial carbon stocks, along with the methodological improvements in the 2019 Refinement. Different carbon conversion factors and the exclusion of other industrial roundwood in the 2019 Refinement also contributed to the different results.

The default method for the production approach is conservative in that it leads to systematically underestimating the global carbon stocks and removals for HWPs, because it confines accounting to products derived from domestic harvest and uses the share of domestic feedstock for accounting. The PA13 in the 2013 Guidance, which is the common approach under the Kyoto Protocol, further reduced the global carbon stocks and resulted in 15% lower carbon removals than those under the PA in the 2006 Guidelines for 2018. The PA and PA13 mainly differ in their handling of the exported feedstock and carbon conversion factor. The PA13i, whose initial year for estimating carbon stocks changed compared to that under the PA13, decreased carbon removals by 27% compared to the PA for 2018. Additionally, the PA19 provided the lowest global carbon removals—45% lower than those under the PA in 2018—mainly owing to the differences in carbon stocks in the initial year. Since the UNFCCC had not stipulated that countries should choose one among the three IPCC guidelines, it is possible that each country could select any of the IPCC guidelines and the corresponding PA, PA13, or PA19, which would lead to considerable removal and emission gaps at the global level.

Previous studies have focused on discussing the various accounting approaches. However, we highlighted that general methodological revisions (e.g., initial years, carbon conversion factors), as well as seemingly small changes in calculation formulas (especially for the production approach), have higher effects than the choice of an approach (i.e., between PA, SCA, and AFA). It should also be noted that the estimated results would change when higher tier methods, compared to the default ones in the respective IPCC guidelines, are applied.

## Methods

### Regional and temporal scope

This study covers all 235 countries and regions available from the forestry module of the FAOSTAT database [43] from 1900 to 2019.

### Differences and similarities between the approaches in the IPCC guidelines

The SCA, AFA, and PA were described in the 2006 Guidelines [12], the PA13 was introduced in the 2013 Guidance [27], and the SCA19, AFA19, and PA19 were indicated in the 2019 Refinement [38]. In our analysis, we apply these approaches, which we consider the most likely choices of countries and the IPCC guidelines for their reporting under the Paris Agreement.

To calculate the HWP stocks and emissions/removals, we follow the respective IPCC guidelines ([12] in the case of SCA, AFA, and PA; [27] in the case of PA13; and [38] in the case of SCA19, AFA19, and PA19). The IPCC guidelines [12, 27, 38] suggest that one of three methods (Tiers 1–3) should be followed. Both the Tier 1 method in the 2006 Guidelines and the 2019 Refinement and the Tier 2 method in the 2013 Guidance apply first-order functions as one of the simplest ways to describe decay processes by using exponential functions, default carbon conversion factors and half-lives of HWPs, and activity data on the HWPs obtained from the FAOSTAT database [43] for each country. To maintain consistency across countries, we used the Tier 1 method in the 2006 Guidelines for the SCA, AFA, and PA and the 2019 Refinement for the SCA19, AFA19, and PA19, as well as the Tier 2 method in the 2013 Guidance for the PA13 as default methods for the respective guidelines. The details of these default methods are explained in this section, as well as in Table 1, emphasizing their differences and similarities. Although the simple-decay approach is also described in the 2006 Guidelines (and the 2019 Refinement), it is excluded from our analysis, as the default (Tier 1) method relevant for the approach is the same as for the PA (and the PA19) [12, 38]. The carbon stock changes in forests, which are also included in the respective IPCC method descriptions, are disregarded in this study. This is because the study looks into the differences in carbon removals/emissions due to differences in the accounting approaches in the IPCC guidelines for HWPs and found that forest carbon stocks would be the same regardless of the approach. We also excluded changes of carbon stocks in waste wood in landfills from our analysis because treatment of waste wood in solid waste disposal sites (SWDSs) is inconsistent in the three IPCC guidelines: waste wood in SWDSs is treated in the waste sector in the 2006 Guidelines and the 2019 Refinement, while it is accounted as instantaneous oxidation in the 2013 Guidance. The following descriptions adopted from Pingoud et al. [6] and the IPCC [12, 27, 38] explain the core differences and similarities between approaches.

The SCA and SCA19 aim to estimate the actual changes in the carbon stocks of wood products within the boundaries of the reporting country, as wood products are accounted for in the country they are used. Assuming that the carbon in wood products is immediately oxidized when these products are discarded, carbon emissions/removals correspond to annual net stock changes, that is, the difference between stocks in the considered year and the subsequent year. The SCA and SCA19 use different half-lives, initial year for carbon stocks, targeted HWPs, and carbon conversion factors (see Table 1).

The AFA and AFA19 are based on the same rationale regarding the carbon stocks of wood products, but the stock changes due to imports/exports are not considered for removals/emissions. Hence, the “carbon burden” of the wood material that is ultimately released into the atmosphere is shifted from exporting to importing countries. For exports and imports, all wood-based materials are considered in the AFA, while only feedstock for wood products are included in the AFA19. Additionally, the AFA and AFA19 use different half-lives, initial year for carbon stocks, targeted HWPs, and carbon conversion factors (Table 1).

The PA, PA13, and PA19 follow the accounting for products made from wood harvested in the reporting country. As such, these guidelines do not estimate the actual carbon stock in the reporting country, but the stock of products made from domestically harvested wood. The PA, PA13, and PA19 use different methods for calculating the share of domestic feedstock, half-lives, initial year for carbon stocks, targeted HWPs, and carbon conversion factors (Table 1).

### Implementation of the IPCC guidelines

The carbon removals by HWPs in each country and for each approach were estimated using Eqs. (1)–(3):

1 $$CR_{SCA/SCA19} (i) = \Delta C_{DC} (i),$$

2 $$CR_{AFA/AFA19} (i) = \Delta C_{DC} (i) + E(i) \, {-}I(i),$$

3 $$CR_{PA/PA13/PA19} (i) = \Delta C_{DH} (i),$$

where *CR*_*SCA*/*SCA19*_(*i*), *CR*_*AFA*/*AFA19*_(*i*), and *CR*_*PA*/*PA13*/*PA19*_(*i*) (tC year^−1^) are the carbon removals during year *i* under the SCA (and SCA19), AFA (and AFA19), and PA (and PA13/PA19), respectively. *∆C*_*DC*_(*i*) (tC year^−1^) is the change in the carbon stocks in HWPs consumed in a country during year *i*. *E*(*i*) (tC year^−1^) is the carbon transfer in the form of wood-based materials exported from the country during year *i*, while *I*(*i*) (tC year^−1^) is the carbon transfer in the form of wood-based materials imported into the country during year *i*. Note that the AFA covers all wood-based materials, including HWPs and feedstock for the HWPs [12], while the AFA19 targets only feedstock for the HWPs [38]. *∆C*_*DH*_(*i*) (tC year^−1^) is the change in the carbon stocks in the HWPs that originate from a country’s domestic harvest during year *i*.

For the SCA, SCA19, AFA, and AFA19, we estimated the changes in the carbon stocks of domestically consumed products (Eqs. (1) and (2)) using the first-order decay functions described in Eqs. (4)–(6):

4 $$\Delta C_{DC/DH} (i) = C_{DC/DH} (i + 1) \, - C_{DC/DH} (i),$$

5 $$C_{DC/DH} (i + {1}) = e^{ - k} \cdot C_{DC/DH} (i) + \, \left[ {({1} - e^{ - k} )/k} \right] \, \cdot Inflow_{DC/DH} (i),$$

6 $$k = {\text{ ln}}\left( {2} \right)/HL,$$

where *C*_*DC*_(*i*) (tC) represents the carbon stocks in the HWPs consumed in the country at the beginning of year *i*. *C*_*DH*_(*i*) (tC) is the carbon stocks in the HWPs derived from domestic harvest at the beginning of year *i*. *Inflow*_*DC*_(*i*) (tC year^−1^) is the carbon inflow to the carbon stocks in HWPs in the form of domestically consumed wood products during year *i*. *Inflow*_*DH*_(*i*) (tC year^−1^) is the carbon inflow to the carbon stocks in HWPs in the form of wood products derived from domestic harvest during year *i*. *k* (year^−1^) is the decay constant of the first-order decay function and *HL* (year) is the half-life for HWPs.

For the SCA and AFA, the initial year in carbon stocks was suggested to be 1900 and the carbon stocks in that year (*C*_*DC*_(1900)) were assumed to be 0, according to the 2006 Guidelines. From 1961 to 2019, *Inflow*_*DC*_(*i*) was calculated using production, import, and export data for each HWP category for each country from the FAOSTAT database [43] to determine domestic consumption (= production + imports − exports). For countries that became independent after 1961 and do not have data before independence in the FAOSTAT database, more recent production, import, and export data were extended back to 1961 using the rate of annual change in each dataset for the old country that the newly independent country was a part of [12] (see Additional file 1). From 1900 to 1961, *Inflow*_*DC*_(*i*) was estimated using Eq. (7):

7 $$Inflow_{DC/DH} (i) = Inflow_{DC/DH} (1961) \cdot e^{{U\left( {i - {1961}} \right)}} ,$$

where *U* represents the annual rates of increase in the HWP consumption by world region from 1900 to 1961, as per the guidelines [12].

For the SCA19 and AFA19, the default method in the 2019 Refinement suggested 1990 as the initial year for carbon stocks, and that the equation with the average value of *Inflow*_*DC*_(*i*) be used over the first five years from 1990 (1990 to 1994). Although the 2019 Refinement also suggested 1961 as the initial year, it simultaneously requested to verify that any historical trends in the relevant statistics for wood commodities reflect actual changes, rather than changes in the coverage of these statistics. In addition, it pointed out the uncertainties associated with the estimate by using earlier initial years (e.g., 1961) [38] (see section "Global perspective"). Therefore, this study applied 1990 as the initial year for carbon stocks and estimated the carbon stocks in that year (*C*_*DC*_(1990)) by using Eq. (8):

8 $$C_{DC/DH} (1990) = \left[ {\mathop \sum \limits_{i = 1990}^{1994} Inflow_{DC/DH} (i)} \right]/5k,$$

In the case of the PA, PA13, and PA19, we calculated the changes in the carbon stocks in the HWPs derived from the domestic harvest (*∆C*_*DH*_(*i*) in Eqs. (3) and (4)) by using *Inflow*_*DH*_(*i*) in Eqs. (5)–(9):

9 $$Inflow_{DH} (i) = P(i) \cdot D_{D/W/P/R} (i),$$

where *P*(*i*) (tC year^−1^) is the carbon from the production of HWPs during year *i*. *D*_*D*/*W*/*P*/*R*_(*i*) is the share of feedstock for the production of HWPs originating from domestic forests during year *i*. We assumed *D*_*D*/*W*/*P*/*R*_(*i*) to be 0 when it took a negative value, according to the guidelines [27, 38].

For the PA, the share of domestic feedstock (*D*_*D*_(*i*) in Eq. (9)) was estimated using Eq. (10):

10 $$ \begin{aligned}D_{D} \left( i \right) & = IRW_{DP} \left( i \right)/\left[ {IRW_{DP} \left( i \right) + IRW_{IM} \left( i \right)}\right.\\&\quad\left.{ \,  {-}IRW_{EX} \left( i \right) + WCP_{IM} \left( i \right){-}WCP_{EX} \left( i \right)}\right.\\&\quad\left.{ + WR_{IM} \left( i \right) \, {-}WR_{EX} \left( i \right)} \right],\end{aligned} $$

where *D*_*D*_(*i*) is the share of feedstock for the production of HWPs originating from domestic forests during year *i*. *IRW*_*DP*_(*i*), *IRW*_*IM*_(*i*), and *IRW*_*EX*_(*i*) (tC year^−1^) refer to the carbon in production, imports, and exports of industrial roundwood (FAOSTAT categories), respectively, during year *i*. *WCP*_*IM*_(*i*) and *WCP*_*EX*_(*i*) (tC year^−1^) denote the carbon in imported wood chips and particles and exported wood chips and particles, respectively. *WR*_*IM*_(*i*) and *WR*_*EX*_(*i*) (tC year^−1^) are the carbon in imported and exported wood residues, respectively.

Under the PA13, the calculation of the carbon inflow was divided into wood and paper product components (*Inflow*_*DH*_ = *Inflow*_*DH*,*wood*_ + *Inflow*_*DH*,*paper*_). The share of domestic feedstock (*D*(*i*)_*W*/*P*_ in Eq. (9)) was estimated using Eq. (11) for sawnwood and wood-based panels and Eq. (12) for paper and paperboard (note the subtraction of exports in the numerator):

11 $$ \begin{aligned}D_{W} \left( i \right) & = \left[ {IRW_{DP} \left( i \right){-}IRW_{EX} \left( i \right)} \right]/\left[ {IRW_{DP} \left( i \right) }\right.\\&\quad\left.{+ IRW_{IM} \left( i \right){-}IRW_{EX} \left( i \right)} \right],\end{aligned} $$

12 $$ \begin{aligned}D_{P} \left( i \right) \, & = \, \left[ {IRW_{DP} \left( i \right) \, {-}IRW_{EX} \left( i \right)} \right]/\left[ {IRW_{DP} \left( i \right) \, }\right.\\&\quad \left.{+ IRW_{IM} \left( i \right) \, {-}IRW_{EX} \left( i \right)} \right] \, \\&\quad \cdot \, \left[ {WP_{DP} \left( i \right) \, {-}WP_{EX} \left( i \right)} \right]/\left[ {WP_{DP} \left( i \right) \, }\right.\\&\quad \left.{ + WP_{IM} \left( i \right) \, {-}WP_{EX} \left( i \right)} \right],\end{aligned} $$

where *D*_*W*_(*i*) is the share of feedstock for the production of sawnwood and wood-based panels originating from domestic forests during year *i*. *D*_*P*_(*i*) is the share of feedstock for the production of paper and paperboard derived from domestic forests during year *i*. *WP*_*DP*_(*i*), *WP*_*IM*_(*i*), and *WP*_*EX*_(*i*) (tC year^−1^) are the carbon in the production, imports, and exports of wood pulp, respectively, during year *i*.

Under the PA19, the share of domestic feedstock (*D*_*W*/*R*_(*i*) in Eq. (9)) was estimated using Eq. (11) for sawnwood and wood-based panels and Eq. (13) for paper and paperboard, which was revised to include recovered paper:

13 $$\begin{aligned} D_{R} \left( i \right) \, =& \, \left[ {IRW_{DP} \left( i \right) \, {-}IRW_{EX} \left( i \right)} \right]/\left[ {IRW_{DP} \left( i \right) \, + IRW_{IM} \left( i \right) \, {-}IRW_{EX} \left( i \right)} \right] \, \cdot \, \{ {1 }{-} \, [RP_{DP} \left( i \right) \\ &\; + RP_{IM} \left( i \right) \, {-}RP_{EX} \left( i \right)\left] / \right[RP_{DP} \left( i \right) \, + RP_{IM} \left( i \right) \, {-}RP_{EX} \left( i \right) + \, WP_{DP} \left( i \right) \, + WP_{IM} \left( i \right) \, {-}WP_{EX} \left( i \right)]\} \, \\ &\;\cdot\left[ {WP_{DP} \left( i \right) \, {-}WP_{EX} \left( i \right)} \right]/\left[ {WP_{DP} \left( i \right) \, + WP_{IM} \left( i \right) \, {-}WP_{EX} \left( i \right)} \right] \, + \, \left[ {RP_{DP} \left( i \right) \, + RP_{IM} \left( i \right){-}RP_{EX} \left( i \right)} \right] \, \\ &\;/\left[ {RP_{DP} \left( i \right) \, + RP_{IM} \left( i \right){-}RP_{EX} \left( i \right) + \, WP_{DP} \left( i \right) \, + WP_{IM} \left( i \right) \, {-}WP_{EX} \left( i \right)} \right] \, \cdot \, \left[ {RP_{DP} \left( i \right){-}RP_{EX} \left( i \right)} \right] \, \\& \;/RP_{DP} \left( i \right) \, + RP_{IM} \left( i \right){-}RP_{EX} \left( i \right)], \\ \end{aligned}$$

where *D*_*R*_(*i*) is the share of feedstock for the production of paper and paperboard derived from domestic forests during year *i*. *RP*_*DP*_(*i*), *RP*_*IM*_(*i*), and *RP*_*EX*_(*i*) (tC year^−1^) are the carbon in the domestic supply, imports, and exports of recovered paper, respectively, during year *i*.

For the PA and PA13, 1900 was the initial year for the carbon stocks in Eq. (7) by using *Inflow*_*DH*_(*i*). Additionally, the 2013 Guidance [27] suggested that the PA13 use another method of estimating the carbon stocks in the initial year by using Eq. (8), based on the average of *Inflow*_*DH*_(*i*) over the first 5 years for which statistical data are available. Therefore, in the modified version of the PA13 (i.e., PA13i), we also used 1961 as the initial year, which was the first year in the FAOSTAT database [43] (see Table 1) and applied Eq. (8) based on *Inflow*_*DH*_(*i*) between 1961 and 1965. Meanwhile, the PA19 used 1990 as the initial year and applied Eq. (8) using *Inflow*_*DH*_(*i*).

## Supplementary Information

**Additional file 1.** HWP activity data. Production, import, and export data for each wood category for each country from the FAOSTAT database.

**Additional file 2.** SCA. National carbon stocks and removals of HWPs for the various countries using the default (Tier 1) method for the stock-change approach according to the 2006 Guidelines.

**Additional file 3.** AFA. National carbon stocks and removals of HWPs for the various countries using the default (Tier 1) method for the atmospheric flow approach according to the 2006 Guidelines.

**Additional file 4.** PA. National carbon stocks and removals of HWPs for the various countries using the default (Tier 1) method for the production approach according to the 2006 Guidelines.

**Additional file 5.** PA13. National carbon stocks and removals of HWPs for the various countries using the default (Tier 2) method for the production approach according to the 2013 Guidance.

**Additional file 6** SCA19. National carbon stocks and removals of HWPs for the various countries using the default (Tier 1) method for the stock-change approach according to the 2019 Refinement.

**Additional file 7.** AFA19. National carbon stocks and removals of HWPs for the various countries using the default (Tier 1) method for the atmospheric flow approach according to the 2019 Refinement.

**Additional file 8.** PA19. National carbon stocks and removals of HWPs for the various countries using the default (Tier 1) method for the production approach according to the 2019 Refinement.

## Data Availability

Datasets are presented in the additional files.

## Abbreviations

HWP: Harvested wood products
SCA: Stock-change approach in the 2006 IPCC Guidelines for National Greenhouse Gas Inventories
SCA19: Stock-change approach in the 2019 Refinement to the 2006 IPCC Guidelines for National Greenhouse Gas Inventories
AFA: Atmospheric flow approach in the 2006 IPCC Guidelines for National Greenhouse Gas Inventories
AFA19: Atmospheric flow approach in the 2019 Refinement to the 2006 IPCC Guidelines for National Greenhouse Gas Inventories
PA: Production approach in the 2006 IPCC Guidelines for National Greenhouse Gas Inventories
PA13: Production approach in the 2013 Revised Supplementary Methods and Good Practice Guidance Arising from the Kyoto Protocol
PA19: Production approach in the 2019 Refinement to the 2006 IPCC Guidelines for National Greenhouse Gas Inventories
UNFCCC: United Nations Framework Convention on Climate Change
COP24: 24Th session of the Conference of the Parties
COP21: 21St session of the Conference of the Parties
CMP7: 7Th session of the Conference of the Parties serving as the meeting of the Parties to the Kyoto Protocol
GHG: Greenhouse gas
IPCC: Intergovernmental Panel on Climate Change
GtC: Gigatons of carbon (1 Gt = 10^9^ tons = 1 Pg)
MtC: Megatons of carbon (1 Mt = 10^6^ tons = 1 Tg)
FAOSTAT: Database of the Food and Agriculture Organization of the United Nations
